# The Effect of Cochlear Implants on Cognitive Function in Older Adults: Initial Baseline and 18-Month Follow Up Results for a Prospective International Longitudinal Study

**DOI:** 10.3389/fnins.2019.00789

**Published:** 2019-08-02

**Authors:** Julia Sarant, David Harris, Peter Busby, Paul Maruff, Adrian Schembri, Richard Dowell, Robert Briggs

**Affiliations:** ^1^Department of Audiology and Speech Pathology, The University of Melbourne, Melbourne, VIC, Australia; ^2^Department of Economics, The University of Melbourne, Melbourne, VIC, Australia; ^3^Cogstate Ltd., Melbourne, VIC, Australia; ^4^The Royal Victorian Eye and Ear Hospital, Melbourne, VIC, Australia

**Keywords:** cognitive decline, hearing loss, cochlear implants, executive function, visual attention, education, age, speech perception

## Abstract

In older adults, hearing loss is independently associated with an increased rate of cognitive decline, and has been identified to be a modifiable risk factor for dementia. The mechanism underlying the cognitive decline associated with hearing loss is not understood, but it is known that the greater the hearing loss, the faster the rate of decline. It is unknown whether remediation of hearing loss with hearing devices can delay cognitive decline. This 5-year international longitudinal study is investigating the impact of cochlear implants on cognitive function in older people with severe-profound hearing loss, and whether remediation of hearing loss could delay the onset of cognitive impairment. This is the first study to examine the major primary risk factors associated with dementia in the same cohort. Participants were assessed before cochlear implantation and 18 months later using an identical battery including a visually presented cognitive assessment tool (Cogstate battery) that is highly sensitive to small changes in cognition and suitable for use with people with hearing loss. Hearing and speech perception ability were assessed in sound-treated conditions by an audiologist, and a range of questionnaire tools was administered to assess self-perceived ease of listening, quality of life, physical activity, diet, social and emotional loneliness, isolation, anxiety, and depression. A detailed medical health history was taken. Pre-operatively, despite the small initial sample size (*n* = 59), increased hearing loss and age predicted significantly poorer executive function and visual attention, while tertiary education predicted better executive function. Better self-reported quality of life was correlated with better visual learning performance, and engaging in frequent vigorous physical activity was correlated with poorer visual learning performance. At 18 months, for the first 20 participants, significant benefits of cochlear implants were seen in terms of speech perception, communication ability, and quality of life. Multiple linear regression modeling showed executive function improved significantly for non-tertiary educated males, while cognitive function remained stable for other participants. Further follow-up at 18 month intervals with a larger sample will reveal the effects of cochlear implant intervention on all outcomes, and whether this can delay cognitive decline.

## Introduction

The prevalence of hearing loss increases with age. In people aged over 65 years it is 30–60%, and increases to 70–90% in people aged over 85 years (Cruickshanks et al., 1998; Sindhusake et al., 2001; Amieva et al., 2015). In older adults (i.e., >65 years) the negative impact of hearing loss on quality of life is substantial, with population norms showing that any form of hearing disability results in poorer physical and mental health outcomes. People with a greater degree of hearing loss are the most affected (Hogan et al., 2009; World Health Organization [WHO], 2009; Swan, 2010). A study of the impact of hearing loss on physical health in older people found that hearing loss was rated the third most problematic condition, after chronic pain and restricted physical activity (Hogan et al., 2009). Hearing loss also causes poorer quality of life, with social, emotional and communication difficulties of increasing magnitude the greater the degree of the hearing loss (Bryant and Sonerson, 2006; Hogan et al., 2009). Communication difficulties often lead to communication breakdown and resulting social and emotional isolation (Wilson et al., 1998) and loneliness (Heine and Browning, 2002; Arlinger, 2003). Social isolation is a form of chronic stress that has been shown to cause depression, poorer cognitive function and poorer overall quality of life (Gopinath et al., 2009; World Health Organization [WHO], 2009; Huang et al., 2010). The World Health Organization has identified three characteristics to define healthy aging: participation in society, physical and mental health, and security. Hearing loss in many cases prevents people from achieving the first two of these characteristics.

Cognitive impairment and dementia in older people with or without hearing loss are also common. Age-related cognitive impairment can progress with time leading to functional disability and loss of independence, both of which increase the burden of caregiving for families and often necessitate institutionalization. After age 65 years, the risk of cognitive impairment is doubled every 5 years, with 3–12% of people aged 70–80 years affected, and 25–35% of people older than 85 years affected (Chen et al., 2009). Cognitive impairment has also been associated with decreases in sensory acuity, particularly as a consequence of hearing loss (Mulrow et al., 1990b). In 2010, there were 35.5 million people worldwide living with cognitive impairment or dementia. By 2030, that number is predicted to increase to 65.7 million, and by 2050 to over 131 million (Wimo and Prince, 2010; Prince et al., 2015). Although some recent reports estimate a slight reduction in the expected incidence of dementia for younger generations (Matthews et al., 2013), this may be balanced by increasing survival, and given the predicted exponential rise in dementia rates over the next few decades, it is expected that the cost of caring for those affected will escalate considerably. Cognitive impairment and dementia will therefore be a significant health problem in the future. In 2009, the global cost of cognitive impairment was estimated at USD422 billion (Wimo et al., 2010), in 2015 USD818 billion, and in 2050 is expected to be USD2 trillion (Prince et al., 2015). Understanding the pathways that lead to cognitive impairment and identifying factors that affect this process in older people is therefore an urgent public health priority.

Hearing loss has recently been shown to be associated independently with the rate of cognitive decline for older adults. A recent large-scale population-based study reported that people with hearing loss had a 30–40% accelerated rate of cognitive decline, and a 24% increased risk for incident cognitive impairment over a 6-year period, compared with people who had normal hearing (Lin et al., 2011). The rate of cognitive decline increased with the severity of hearing loss, with every 10 dB of hearing loss associated with faster decline. The first meta-analysis in 2015 of the link between hearing loss and cognitive decline concluded that cognition was significantly poorer in people with untreated hearing loss, with the magnitude of cognitive deficit associated with the degree of hearing loss (Taljaard et al., 2016). The Lancet Commission has recently identified hearing loss as a modifiable risk factor for dementia, contributing 9% of the risk for incident dementia (Livingston et al., 2017). In a systematic review of the impact of cochlear implantation on cognition, a significant difference in cognitive function was also found between people whose hearing loss was ameliorated by cochlear implants and those whose loss remained untreated, however, this conclusion was based on only three studies that included devices that would now be considered obsolete. It was concluded that “further research is required to understand whether hearing impairment is a cause of cognitive deficits…and whether hearing intervention mitigates any effects on cognitive function” given significant methodological limitations in the studies conducted to date.

It is possible that associations between hearing loss and poorer cognitive function could reflect degeneration of a common neuropathologic origin, i.e., both are caused by the degenerative/aging process. A further hypothesis is that for older people with a significant hearing loss, communication difficulties, and the resulting isolation from others and consequent loneliness contribute to cognitive decline. Neuroanatomic (Lindenberger and Baltes, 1994) and epidemiological studies provide support for this theory (Fratiglioni et al., 2000; Bennett et al., 2006), with strong reported associations between loneliness and cognitive decline. Having a limited social network has been found to increase the risk of dementia by 60% (Barnes et al., 2004), while a further study reported having a high level of social engagement reduced cognitive decline by 91% (Cacciatore et al., 1999). Hearing loss has also been found to be associated strongly with depression (Mulrow et al., 1990a; Chia et al., 2007), and depression in turn with cognitive impairment (Forsell et al., 1994), depression even being considered a risk factor for cognitive impairment (Jorm, 2000). As concluded in a recent review of the possible mechanisms of the hearing-cognition relationship (Fulton et al., 2015), these appear to be multiple, and include neuropathic degeneration, sensory degradation/deprivation, increased cognitive load, social isolation and depression, and over diagnosis (as a result of the use of cognitive assessment tools inappropriate for people with hearing loss).

There is some evidence that treatment of hearing loss with hearing aids can significantly reduce depression, anxiety, and loneliness for people with hearing loss, and may improve cognitive function (Dawes et al., 2015; Deal et al., 2015). There is also some evidence that use of cochlear implants can improve quality of life and/or cognitive function, although most of the studies have been short term (Francis et al., 2002; Vermeire et al., 2005; Mosnier et al., 2015, 2018; Manrique-Huarte et al., 2016; Jayakody et al., 2017b; Maharani et al., 2018). However, studies that have examined the effect of treatment of hearing loss on cognition in older adults to date have had numerous and significant methodological limitations that limit the applicability of the findings. For example, many rely on small samples (e.g., Jayakody et al., 2017b; *n* = 16) or use a retrospective design (e.g., Manrique-Huarte et al., 2016). Audiological data on hearing loss was often not obtained, or this was done via self-report, which is known to be highly inaccurate (Gallacher et al., 2012; Mick, 2018). Change in hearing was often not assessed over time, and information about frequency of hearing aid use or benefit was also not collected (Maharani et al., 2018). Further, a number of other factors likely to influence outcomes, such as social participation, mood, exercise, quality of life, diet, hearing aid use, and speech perception, were often not considered. For many studies, as is current standard practice (Dupuis et al., 2015), the assessment of cognitive function was conducted using screening tools for dementia which are not sensitive and are usually administered using verbal instructions, such as the Mini Mental State Examination (Amieva et al., 2015; Mosnier et al., 2015, 2018). However, the use of verbal instructions disadvantages people even with mild-moderate hearing loss, and is therefore likely lead to an overestimation of cognitive deficit (Murphy et al., 2006; Dupuis et al., 2015). The use of such tools also provides no specific information about changes in cognitive function or rates of change over time, and likely underestimates cognitive function for people with hearing loss. These methodological limitations make it difficult to determine whether there is a relationship between rate of hearing loss decline and rate of cognitive decline in the samples in these studies. Further, the effects of degree of hearing loss, device benefit or amount of hearing aid use also could not be established. Therefore, although it is known that faster cognitive decline is associated with greater degrees of hearing loss, the effects of treatment of hearing loss on cognitive function in adults have not yet been investigated in a well-designed study that accurately assesses both hearing loss and cognition and controls for the factors associated with decline in both hearing loss and cognition. As concluded in the 2015 meta-analysis of hearing loss and cognitive decline (Miller et al., 2015), “further research is required to understand…whether hearing intervention mitigates any effects on cognitive function.”

Other factors that are reported to influence the incidence of cognitive decline include sex and education. Although many studies and a meta-analysis of eight European population-based studies have suggested that females are at higher risk of dementia (e.g., Gao et al., 1998; Andersen et al., 1999; Roberts et al., 2012; Chene et al., 2015), many others have reported no difference in incidence (e.g., Prencipe et al., 1996; Ruitenberg et al., 2001; Katz et al., 2012). It is therefore still unclear whether females have a higher risk than males of developing dementia (Rocca et al., 2014). Given the contradictory nature of the published evidence to date, and reported differences in the incidence of cognitive decline according to clinical subtype and sex (e.g., amnestic and non-amnestic mild cognitive impairment), it has been recommended that risk factors for cognitive decline should be investigated separately for males and females (Roberts et al., 2012). Conversely, lower education is widely accepted as one of the most established risk factors for dementia, and therefore is an important predictive factor for cognitive outcomes, although the exact mechanisms through which education is protective of cognitive function are still unknown (Cobb et al., 1995; Ott et al., 1995; Prencipe et al., 1996; Livingston et al., 2017).

The current multi-center study follows cognitive function over time in a prospectively recruited cohort of older recipients of cochlear implants in Australia and New Zealand. The study addresses the methodological limitations of previous studies in conducting rigorous audiological hearing assessments at baseline (prior to implantation) and at all follow-up 18-month intervals. Other factors likely to influence outcomes, such as social isolation and loneliness, mood, exercise, quality of life, diet, and device use are assessed so that their effects of cognitive outcomes can be taken into account. Cognitive ability is measured using a computerized non-auditory tool to avoid the use of verbal instructions, and device use and outcomes (speech perception and ease of listening) are also assessed in order to determine the degree of treatment benefit. Outcomes for this population of older people with the most significant degree of hearing loss will be compared in the future with those of a healthy aging comparison group of older Australians with typical hearing for their age; data collection to enable this is ongoing. The first aim of this study is to investigate the relationship between degree of hearing loss and the extent of cognitive impairment at baseline. The second aim is to examine the effect on cognition of cochlear implantation. The third aim is to determine the extent to which cochlear implantation is associated with changes in quality of life. This study will provide important and world-first rigorous evidence that will inform future policy and clinical practice on the management of older adults around the world with hearing loss. It is critically important to investigate whether treatment of hearing loss with cochlear implants could delay the onset of cognitive impairment in this population of older adults who are most affected by hearing loss.

## Materials and Methods

### Participants

Fifty-nine adults aged 61–89 years, with severe-profound hearing loss (mean better ear PTA 76 dB) and no previously diagnosed or suspected cognitive impairment participated in this study. They had sufficient English to be able to give informed consent, comprehend test instructions and complete questionnaires. The participants were all patients of the Royal Victorian Eye and Ear Hospital Cochlear Implant Clinic, and had been identified as suitable to proceed with cochlear implantation at the time of recruitment. **Table 1** shows participant audiometric and demographic information. Participants received either the CI512, CI522, or CI532 Cochlear Nucleus implants (contour array, slim straight, or slim contour). All participants used the ACE speech processing strategy (Skinner et al., 2002), along with either the Cochlear Nucleus 6 or Nucleus 7 speech processor.

**Table 1:** Demographic and audiometric characteristics of the study sample (*N* = 59).

	All	Male	Female	*p*^∗^
Number	59	36	23	
Age (Mean)	72.3	72.4	72.1	
Age (SD)	6.8	7.5	5.6	
Pre-op PTA better ear (Mean)	76.2	71.4	83.7	**0.011**
Pre-op PTA better ear (SD)	18.7	18.4	16.8	
Pre-op worse ear (Mean)	98.4	96.6	101.2	0.255
Pre-op worse ear (SD)	15.8	16.9	13.7	
Retired (%)	79.3	74.3	87	0.227
Diabetes (%)	12.1	8.6	17.4	0.354
Past smoker (%)	44.8	45.7	43.5	0.87
Cardiac conditions (%)	70.7	71.4	69.6	0.882
Depression (%)	13.8	11.4	17.4	0.544
Anxiety (%)	19	11.4	30.4	0.099
Vigorous activity (%)	31.5	35.5	26.1	0.466
**Highest education level**				
High school (%)	58.6	51.4	69.6	0.17
Undergraduate (%)	13.8	11.4	17.4	0.544
Postgraduate (%)	27.6	37.1	13	0.032

*PTA, pure tone average threshold. ^∗^p-value for test for difference of means between male and female. All bolded values are significant at the 5% level.*

### Procedures

This study was carried out in accordance with the recommendations of the Australian National Health and Medical Research Council guidelines for ethical research conduct. The protocol was approved by the Royal Victorian Eye and Ear Hospital (reference no. 15/1217H). All participants gave written informed consent in accordance with the Declaration of Helsinki. All participants completed a pre-operative (baseline) assessment battery comprising audiometry, speech perception testing, cognitive screening and assessment, and health, quality of life, lifestyle and ease of listening questionnaires. Participants who were assessed at the 18-month post-operative point completed an identical assessment battery, and in addition reported on their device use, data for which was also obtained through the data logging function of their speech processor.

### Audiological Assessment

All participants were assessed pre-operatively by an audiologist in a sound-treated booth, as part of the RVEEH standard pre-operative cochlear implant workup, and again 18 months later by the research team. Audiometric assessment included air and bone conduction thresholds, speech discrimination assessment, and tympanometry. Speech perception ability was assessed in the best aided condition (using hearing aids where possible) using both word and sentence level materials. Consonant-vowel-consonant (CVC) monosyllabic words (50 word lists; scored for words and phonemes correct) were presented at 65 dBSPL in quiet in the left ear, right ear and binaurally. Speech Reception Threshold testing (SRT) was conducted using 20 Bamford-Kowal-Bench-like sentence lists in four-talker babble background noise. The test sentence was presented at 65 dBSPL, while the noise level was adaptive, i.e., the level of noise presented was altered depending on the score for each sentence, with the final measure reflecting the signal-to-noise ratio at which 50% of the key words were identified. Both the target sentence and background noise were presented one meter in front of the participant via a single speaker in the free field. Correctly repeated target words were scored for each sentence, and the mean performance score in signal to noise ratio in decibels was used to calculate the participants’ ability to perceive speech in noise for the right ear, left ear and binaurally. The non-test ear was masked in the unilateral listening conditions using white noise set at 30 dB above the average of the 1 and 2 kHz thresholds.

### Cognitive Assessment

Cognitive screening was conducted for all participants using the Mini Mental State Examination (MMSE; Folstein et al., 1975), a screening test for dementia. In accordance with the latest NICE (2011) guidelines, a cut-off score of 24/30 was used to identify people with cognitive impairment.

Cognitive function was assessed using the Cogstate Brief Battery and the Cogstate Groton Maze Learning Test (GMLT) of executive function (CSBB; Westerman et al., 2001; Collie et al., 2003; Falleti et al., 2006; Maruff et al., 2009) by trained staff both pre-operatively, and 18 months post-operatively. The CSBB is a computerized test battery that has been developed for repeated assessment of cognitive performance. The battery is relatively quick to administer (30 min), highly reliable (test-retest reliability for each measure ranges between 0.84 and 0.94), and facilitates minimal practice effects (Falleti et al., 2006). The CSBB includes tests of visual learning, working memory, attention, and psychomotor function. It can detect decline in cognitive function that is not apparent over even a 6-month period (Lim et al., 2013). This tool is ideal for use with people with hearing loss, as it is visually presented. Both the speed and accuracy of responses are recorded (Falleti et al., 2006; Maruff et al., 2009).

The GMLT assesses executive function, and takes 7 min to administer on average. Using a maze learning paradigm, the total number of errors made when attempting to learn the same hidden pathway across five trials presented consecutively is calculated. Lower scores indicate better performance.

The Detection (DET) Task assesses psychomotor function and takes 3 min to administer on average. The participant must respond “yes” when a card in the center of the screen turns over until 25 correct responses are obtained, or the maximum time limit (2 min) has been reached (whichever occurs first). Performance speed (milliseconds) taken to complete the task is recorded. Lower scores indicate better performance.

The Identification (IDN) Task assesses visual attention and takes 3 min to administer on average. A choice reaction paradigm in which the participant must answer “yes” or “no” to the question “Is the card red?” when a playing card in the center of the screen turns over is used. Performance speed (milliseconds) to complete the task is recorded. Lower scores indicate better performance.

The One Card Learning (OCL) Task assesses visual learning and takes 6 min to administer on average. A pattern separation paradigm in which the participant must answer “yes” or “no” to the question “Have you seen this card before in this test?” when a playing card in the center of the screen turns over is used. The test measures performance accuracy. Higher scores indicate better performance.

The One Back (ONB) Task assesses working memory and takes 4 min to administer on average. Working memory is assessed using an n-back paradigm in which the participant must answer “yes” or “no” to the question “Is the previous card the same?” when a playing card in the center of the screen turns over. Both the speed and accuracy of performance are measured. Lower scores indicate better performance.

Composite scores were also calculated (Processing speed and Memory composite) using normalized *Z* scores (Falleti et al., 2006; Maruff et al., 2009).

### Medical Health History

A detailed health history, including medical history was taken, (including family history of mental and other neurological illnesses), along with a personal health history, including smoking, current and past alcohol use, illicit drug and medication use.

### Quality of Life Measures

#### Anxiety and Depression

The Hospital Anxiety and Depression Scale (HADS; Zigmond and Snaith, 1983) measures levels of depressive and anxiety symptoms. This tool generates ordinal data, and was designed for use with people who have physical health problems. For anxiety, specificity is 0.78, with a sensitivity of 0.9, and for depression, 0.79 and 0.83, respectively.

#### HUI-3 Quality of Life

Health status and health-related quality of life (HRQOL) for all participants was assessed using the Health Utilities Index-3 Quality of Life Questionnaire (HUI-3; Horsman et al., 2003), as one means of measuring CI benefit. The HUI-3 measures vision, hearing, speech, ambulation, dexterity, emotion, cognition, and pain. It has been a reliable, responsive and valid measure in a number of clinical studies. Utility scores provide an overall assessment of the HRQOL of patients.

#### Ease of Listening/Subjective Device Benefit

Ease of listening and difficulties in everyday communication situations were measured using the Abbreviated Profile of Hearing Aid Benefit (APHAB; Cox and Alexander, 1995), a questionnaire designed to measure self-reported auditory disability in everyday living. The scale covers hearing speech in a variety of competing contexts and different everyday sounds across four subscales: Ease of Communication, Reverberation, Background Noise, and Aversiveness of sounds. Higher scores indicate better performance, with the exception of Aversiveness, where a higher score indicates increased aversiveness.

#### Health and Lifestyle

Two questionnaires were used to assess health and lifestyle.

The International Physical Activity Questionnaire (IPAQ; long form, 31 items; Craig et al., 2003) monitors population levels of physical activity and inactivity in adults. It has four domains: (1) during transportation, (2) at work, (3) during household and gardening tasks, and (4) during leisure time, including exercise and sport participation.

The Bayer Activities of Daily Living Scale (Hindmarch et al., 1998) was used to assess difficulties in the performance of everyday activities. The 25-item scale was completed by a caregiver or person familiar with the participant. The items in the scale are sensitive to cognitive impairment, are simple in concept, and reflect a wide range of domains. Lower scores indicate less difficulty.

### Loneliness and Social Participation

The Lubben Social Network Scale (LSNS; Lubben, 1988) is a brief instrument designed to gauge social isolation in older adults by measuring perceived support received from family and friends. It typically takes 5–10 min to complete and comprises an equally weighted number of items used to measure size, closeness and frequency of a respondent’s social network.

The Loneliness Scale (de Jong-Gierveld and Kamphuls, 1985) is an 11-item scale designed to measure subjective feelings of loneliness as well as feelings of social isolation. Participants rate each item as either O (“I often feel this way”), S (“I sometimes feel this way”), R (“I rarely feel this way”), N (“I never feel this way”). Typically, scale reliability in the 0.80–0.90 range is observed.

### Device Use Compliance

In evaluating treatment effects, it is important to measure compliance with device use. All participants who were assessed at the 18-month post-operative point completed a brief questionnaire about their use of their CI, including how many hours per day they used it.

### Statistical Analysis

Summary statistics and correlations are calculated to describe the characteristics of the participants at baseline and the pairwise relationships between the various outcomes. Given the published evidence that cognition in adults is dependent on age, sex, and education level, baseline relationships between cognition, hearing, age, sex, and education were quantified using regression. For a cognition score *Y* (which could be one of GML, IDN, OCL, ONB, or OCL), the regression has the form:

(1) $$Y=\beta_{0}+\beta_{1}\;\mathrm{BPTA}+\beta_{2}\;\mathrm{Age}+\beta_{3}\;\mathrm{Female}+\beta_{4}\;\mathrm{UGrad}+\beta_{5}\;\mathrm{PGrad}+U$$

where BPTA is the PTA hearing loss in the better ear measured in units of 10 dB, Age is age in years, Female = 1 for a female participant and 0 otherwise, UGrad = 1 if years of education is 13–15 years and 0 otherwise, and PGrad = 1 if years of education is 16 years or greater and 0 otherwise. *U* represents the regression error. The interpretation of β_1_ is that an increase of 10 dB in better ear hearing loss corresponds to a change of β_1_ in the mean of *Y*, controlling for age, sex, and educational level.

Average changes from baseline to 18 months in cognition, quality of life, loneliness and social participation, physical activity, and ease of listening were calculated and tested for significance. Changes in the key cognitive and quality of life outcomes were also analyzed in more detail in terms of age, sex and education using the regression:

(2) $$\mathrm{DY}=\gamma_{0}\;\mathrm{Male}+\gamma_{1}\;\mathrm{Female}+\gamma_{2}\;\mathrm{Higher Ed.}+\gamma_{3}\;\mathrm{Age}70+U$$

where *DY* is the change in outcome *Y* (any cognitive or HUI score) from baseline to 18 months, Male and Female are 0/1 indicators as described above, and Higher Ed. is the combination of the UGrad and PGrad indicators in Eq. (1) (the combination being necessary because of the smaller sample size of 18 month observations available), and Age 70 is age in years above or below 70. For example, the average of *DY* for females of age 70 with fewer than 13 years education would be given by γ_1_.

## Results

### Baseline

A summary of the baseline characteristics of the participants (overall, and by sex) is presented in **Table 1**. Average better ear hearing loss was greater for females (83.7 dB) than for males (71.4 dB), a difference that was statistically significant (*p* = 0.011).

Pairwise correlations between cognition scores and other measurements are presented in **Table 2**. There was a positive correlation (*r* = 0.280) between baseline better ear hearing loss and the GMLT where greater hearing loss was associated with poorer executive function. The APHAB Global score, Ease of Communication score, Reverberation score and Aversiveness score were all negatively associated with GMLT scores – better APHAB scores were associated with better executive function. Although only three participants had anxiety at baseline, greater anxiety was also positively correlated with poorer executive function (*r* = 0.31). Only one participant had results consistent with depression. Better self-reported quality of life (HUI-3) was also moderately positively correlated with improved visual learning performance (0.40), and engaging in frequent vigorous physical activity was moderately correlated with poorer visual learning performance (−0.30).

**Table 2:** Pairwise correlations.

	GML	IDN	OCL	ONB	DET	PS	MC	BPTA
Past smoking years	−0.03	−0.19	−0.07	−0.02	0.08	0.02	−0.03	−0.01
Memory complaints score	−0.19	−0.19	0.11	−0.30	−0.12	0.16	0.27	−0.04
HUI3 – total scores	−0.19	0.14	0.40	0	−0.05	−0.11	0.24	0.01
HUI3 hearing disability score	−0.12	0.19	0.11	0.12	−0.02	−0.15	−0.03	−0.08
IPAQ activity – total score	0.04	0.03	−0.23	0.08	0.02	−0.04	−0.25	−0.09
IPAQ activity – walking	0.06	−0.02	−0.15	0.11	−0.14	0.08	−0.23	−0.14
IPAQ activity – moderate	0.11	0.15	−0.12	0.06	0.17	−0.16	−0.12	0.09
IPAQ activity – vigorous	−0.12	−0.15	−0.30	0	−0.08	0.08	−0.27	−0.26
IPAQ activity – sitting	−0.05	−0.20	0	−0.23	−0.05	0.13	0.17	0.05
LSNS Loneliness Scale – total score	0.16	−0.11	0.11	0.14	0.07	0.04	0.01	0.03
Lubben Loneliness Scale – total score	0.03	0.24	−0.06	0.14	0	−0.12	−0.13	0.18
Bayer conversation score	−0.12	0.09	−0.10	−0.13	−0.10	0.03	0.03	0.13
Bayer telephone score	0.15	0.10	0.01	0.07	0	−0.06	−0.02	0.02
APHAB, Global score	−0.52	0.09	0.08	0.06	−0.10	−0.03	−0.01	−0.08
APHAB, Ease of communication	−0.53	0.19	0.17	0	−0.12	−0.04	0.12	0.02
APHAB, Background noise	0.39	0.08	−0.09	0.12	0.03	−0.11	−0.20	−0.08
APHAB, Aversiveness	−0.35	0.15	0.16	0.03	−0.05	−0.05	0.11	−0.07
Speech perception – CVC word score, CI ear	−0.27	−0.09	−0.11	0.06	0.04	−0.01	−0.14	−0.75
Speech perception – CVC phoneme score, CI ear	−0.32	−0.09	−0.12	0.04	0.01	0	−0.14	−0.74
Speech perception in noise – SRT score, CI ear	0.26	0.18	0.04	−0.09	0	−0.07	0.11	0.66
Depression	−0.03	−0.09	−0.07	0.02	−0.14	0.13	−0.07	−0.24
Anxiety	0.31	−0.01	−0.05	0.06	−0.12	0.04	−0.12	0.07
Better ear PTA (pre-op)	0.28	0.17	0.07	−0.04	0.01	−0.07	0.1	

*PS, processing speed composite score; MC, memory composite score; BPTA, better ear pure tone average hearing threshold.*

**Table 3** presents results of regression models for the primary outcome scores for the five aspects of cognition as functions of hearing, age, sex, and education level. The table shows coefficient estimates (standard errors in brackets) for Eq. (1) for each of the five cognition outcomes, along with *R*^2^ and the mean and standard deviation of each outcome. The results show a statistically significant increase (i.e., deterioration) in the mean GMLT scores of 5.663 points for every 10 dB increase in hearing loss (PTA) in the better ear, controlling for age, sex, and education. The magnitude of this change was 9.2% of the mean GML outcome. Also, the mean GMLT score was 19.915 points lower (i.e., better) for participants with at least 16 years of education, a difference of 32.5% of the overall mean. The regression explains 34.1% of the variation in GMLT scores. The regression for ONB contains two marginally statistically significant but practically non-significant coefficients.

**Table 3:** Regressions for baseline cognitive scores.

	GML	IDN	OCL	ONB	DET
Intercept	10.401	2.694^∗^	0.963^∗^	2.917^∗^	2.563^∗^
	(15.631)	(0.040)	(0.067)	(0.049)	(0.045)
BPTA	**5.663^∗^**	0.006	0.002	−0.002	0.002
	(1.976)	(0.005)	(0.008)	(0.006)	(0.006)
Age	0.998	0.003	−0.003	**0.003^∗^**	0.003
	(0.545)	(0.001)	(0.002)	(0.002)	(0.002)
Female	−1.384	−0.018	0.051	−0.009	−0.03
	(7.746)	(0.019)	(0.032)	(0.024)	(0.022)
Undergraduate Ed.	8.579	0.027	−0.045	**0.072^∗^**	0.003
	(10.071)	(0.026)	(0.043)	(0.032)	(0.029)
Postgraduate Ed.	**−19.915^∗^**	−0.02	0.008	−0.027	−0.035
	(8.528)	(0.020)	(0.034)	(0.025)	(0.024)
*R*^2^	0.341	0.151	0.116	0.2	0.153
**Outcome**					
Mean	61.368	2.766	0.946	2.945	2.591
SD	29.251	0.066	0.112	0.084	0.076

*^∗^Significant at 5% level. Specification: see Eq. (1). BPTA, better ear pure tone average hearing threshold; Age, age in years; Female, 1 if participant was female, 0 otherwise; Undergraduate Ed, 1 if participant had 13–15 years of education, 0 otherwise; Postgraduate Ed, 1 if participant had more than 15 years of education, 0 otherwise. All bolded values are significant at the 5% level.*

### 18 Months Post Switch-On

**Table 4** presents means for the outcomes at baseline and at 18 months, with the mean differences and *p*-values included.

**Table 4:** Mean outcomes at baseline and 18 months (*N* = 20).

	Baseline	18 months	*p*-value^∗^
Hearing – Objective measures			0.000
Speech perception – CVC word score %	9.4	66.3	
Speech perception – CVC phoneme score %	27.4	82.4	
Speech perception – SRT	16.0	6.2	
Hearing – Subjective measures			0.000
APHAB Global	15.19	44.66	
APHAB Ease of communication	18.41	52.49	
APHAB Reverberation	12.76	42.27	
APHAB Background noise	14.41	39.23	
APHAB Aversiveness	−22.12	−16.02	
**Cognition**			
GML	59.17	48.26	
IDN	2.77	2.79	
OCL	0.94	0.93	
ONB	2.95	2.95	
DET	2.61	2.64	
HUI3 total	0.56	0.67	
HUI3 hearing disability	0.38	0.67	
Activity – total	4541.17	5984.76	
Loneliness total	2.64	1.39	
Lubben total	45.36	44.77	
Bayer conversation	5.22	2.83	
Bayer telephone	6.13	5.57	
Depression	2.22	1.96	
Anxiety	4.00	3.13	

*^∗^p-values are from joint tests of equality of means for the multiple outcomes.*

#### Device Use and Benefit

Device use questionnaire responses indicated that participants were using their device on average 14 h per day, with a range of 6.5 h per day (one participant only) up to 16 h per day. All except one participant showed an improvement in speech perception scores in both quiet and noise at 18 months post switch-on. In quiet, the mean CVC word score increased from 9.4 to 66.3% correct, and the mean CVC phoneme score increased from 27.4 to 82.4%. In noise, for those who were able to do SRT testing pre-operatively, the mean SRT score decreased from 16.0 to 6.2, demonstrating an improved ability, on average, to perceive speech in background noise. Considered jointly, these changes in speech perception were highly statistically significant (*p* < 0.0001).

All APHAB scores except for Aversiveness significantly improved from baseline to 18 months. In particular, the Global score improved from an average of 15.19 at baseline to 44.66 after 18 months, almost tripling the baseline average score on this scale of self-reported listening disability in everyday life. The changes in the mean APHAB scores were highly statistically significant (*p* < 0.0001).

The mean difficulty score on the Bayer conversation scale improved substantially, from 5.22 to 2.83, with a reduction in difficulty of 54%.

#### Cognition

Of the cognition scores, the improvement of greatest magnitude was in the GMLT score (18% of baseline mean).

The key cognitive outcomes were analyzed in more detail using regressions of the form of Eq. (2), with results given in **Table 5**. For executive function (GMLT) the average score for males without higher education (i.e., Higher Ed. = 0) changed by −21.949 points (*p* = 0.010), relative to the overall baseline mean of 61.368, indicating an improvement in average executive function. The average GMLT score for males with higher education (i.e., Higher Ed. = 1) changed by −21.949 + 18.768 = −3.181, but this change was not statistically significant. The average GMLT score for females also improved, changing by −11.883 points, but this difference was not statistically significant. There was only one female with higher education so separate results by education for females are not available from this sample. The overall implication is that the average executive function (GMLT) score improved by a statistically and practically significant amount for males with at most 12 years of education, but apart from these less educated males the average GMLT scores were not (statistically) different in this sample.

**Table 5:** Regressions for baseline – 18 months changes.

	GML	IDN	OCL	ONB	DET	HUI-3 total	HUI-3 hearing
Male	**−21.949^∗^**	0.013	−0.019	0.012	−0.01	0.015	0.136
	(7.548)	(0.017)	(0.033)	(0.031)	(0.055)	(0.068)	(0.111)
Female	−11.883	−0.002	−0.044	−0.037	**0.116^∗^**	**0.141^∗^**	**0.338^∗^**
	(6.906)	(0.017)	(0.031)	(0.029)	(0.052)	(0.064)	(0.105)
Higher Ed.	**18.768^∗^**	0.013	0.015	−0.008	−0.002	0.072	0.066
	(8.591)	(0.021)	(0.039)	(0.037)	(0.065)	(0.080)	(0.132)
Age 70	0.846	**0.005^∗^**	0.004	0.000	0.001	0.01	**0.027^∗^**
	(0.733)	(0.002)	(0.003)	(0.003)	(0.005)	(0.007)	(0.011)
*R*^2^	0.379	0.488	0.129	0.109	0.267	0.471	0.683
*n*	20	23	23	23	23	23	23

*^∗^Significant at 5% level. Specification: see Eq. (2). Male, 1 if participant was male, 0 otherwise; Female, 1 if participant was female, 0 otherwise; Higher Ed., 1 if participant had more than 12 years of education, 0 otherwise; Age 70, difference of age in years from 70. All bolded values are significant at the 5% level.*

#### Quality of Life/Mood

As shown in **Table 4**, the mean HUI-3 quality of life scores (total and hearing disability) both improved from baseline to 18 months. The regression results in **Table 5** show that the HUI-3 outcomes for females had statistically significant improvements at 18 months post switch-on. For females, the mean HUI-3 total score increased by 0.141 (*p* = 0.041) relative to the female mean baseline score of 0.466, and the mean HUI-3 hearing disability score increased by 0.338 (*p* = 0.005) relative to the overall mean of 0.274. Significant improvements were not found for males, whose baseline means of 0.596 for the HUI-3 total score and 0.427 for the HUI-3 hearing disability score were higher than for the females (albeit only significant at the 10% level for the total score). These results show that average quality of life for the female participants was improving, with their scores “catching up” to those for the males. The Age variable was statistically significant in the regression for the HUI-3 hearing disability score, implying that older participants showed larger mean improvements in this aspect of quality of life.

At 18 months after cochlear implantation, the number of participants with anxiety had reduced from one to three, and one participant remained depressed.

## Discussion

### Baseline Outcomes

#### Hearing Loss and Cognition

At baseline, regression modeling (controlling for age, sex, and education) predicted significantly poorer executive function with greater hearing loss, with marginal effects modeling predicting that for every 10 dB increase in hearing loss, there would be a corresponding decline in executive function of over 9% of the mean outcome on this measure. As mentioned earlier, a relationship between age-related hearing loss and cognitive function has been reported in several studies of older adults (see Miller et al., 2015) and in a recent systematic review and meta-analysis of both cross-sectional and longitudinal cohort studies (*n* = 15) (Loughrey et al., 2018). Executive function was also reported to be significantly poorer with increasing hearing loss in another recent study of 119 potential CI recipients aged 45–85 years with bilaterally symmetrical mild-profound hearing loss (Jayakody et al., 2017a).

The current study and that of Jayakody et al. (2017a) found that of all the cognitive domains assessed at baseline (i.e., prior to cochlear implantation), executive function was most affected by hearing loss. Cortical auditory evoked potential and neuroimaging studies have shown increases in P2 latency and amplitude and decreased activation of the temporal cortex, even in the early stages of mild-moderate hearing loss, and it has been theorized that short-term memory and executive functions are recruited to assist with the perception of speech in the presence of hearing loss and the associated shrinking of the auditory cortex, thus preserving these functions while others are lost due to reallocation of cognitive resources (Campbell and Sharma, 2013).

A meta-analysis by Loughrey et al. (2018), which included 14 studies that had measured aspects of executive function as well as attention and working memory, concluded that hearing loss was associated less with decline in executive function and impacted more on delayed and semantic memory, although in many studies of older adults, performance on the tests of semantic memory are used as indices of executive function. The differences in findings between the current study and those of the meta-analysis could be due to the types of tests used to assess executive function. Whereas the Cogstate battery utilizes a visual and non-linguistic method of presentation suitable for use with adults with severe-profound hearing loss, has no measurable practice effects, and very high sensitivity and reliability, no details of the tests used in the studies included in the meta-analysis were provided, with the exception of one screening tool. In addition, information about whether the tasks were verbal or non-verbal was not presented. If verbal executive function tasks were used, there could be issues of confounding due to the inter-relationship between verbal language and executive function, as well as that between hearing loss and comprehension of instructions. Further, while the GMLT from the Cogstate Battery comprehensively assesses multiple aspects of executive function, many of the tests used in the 14 studies covered limited aspects of executive function (e.g., attention only), or used measures of global cognitive function. The latter are insensitive in the absence of significant cognitive impairment and are clearly unsuitable for administration with people with hearing loss (such as the MMSE), which in this study was used only as a screening tool to exclude participants at baseline with clear dementia.

#### Education and Cognition

Lower education has long been identified as a risk factor for dementia, and is thought to contribute to vulnerability to cognitive decline because it results in lower cognitive reserve. Cognitive reserve enables more highly educated people to maintain cognitive function in the presence of brain pathology (Anstey and Christensen, 2000; Livingston et al., 2017). Low educational attainment has been identified as one of the strongest modifiable risk factors for cognitive decline, estimated using relative risks from meta-analyses to contribute over 19% of the population-attributable risk (PAR) of Alzheimer’s disease worldwide (Norton et al., 2014). The fact that education is protective of cognitive function was evident in the baseline finding of this study that executive function was significantly better for participants with at least 16 years of education.

#### Exercise and Cognition

Inactivity has also been identified as a modifiable risk factor for dementia, and was recently the highest estimated PAR in the United States (21%), Europe (20.3%), and the United Kingdom (21.8%) (Norton et al., 2014). Despite this, the literature examining the relationship between exercise and cognition reports mixed findings. Some studies have reported that exercise is effective in maintaining cognitive function and should be considered as a non-pharmaceutical intervention for the prevention of cognitive decline and neurodegenerative diseases (Bherer et al., 2013; Mandolesi et al., 2018). It is thought that a beneficial effect on cognition is due to the effects of exercise on vascular function and on increasing the size of the prefrontal and hippocampal areas of the brain, promoting better working memory (Brown et al., 2010; Davenport et al., 2012; Erickson et al., 2012). However, there is also evidence suggesting that exercise does not appear to promote any improvement in cognitive function in older people either with or without cognitive impairment (Sink et al., 2015; Young et al., 2015). In the current study, vigorous physical activity was correlated with poorer visual learning performance at baseline. This is only one correlation, however, there is some emerging evidence in the recent literature of negative effects of physical over-exertion on cognition in older people, with a recent study suggesting that moderate intensity exercise programs may even increase the rate of cognitive decline in people with dementia (Lamb et al., 2018). As suggested in a systematic review by van Uffelen et al. (2008), further high quality trials of the effect of physical activity on cognition in older adults are required, as the current literature suffers from a lack of high quality studies, with large variability in study populations, exercise protocols, and outcome measures. The current study will continue to monitor the effects of physical activity on cognition over time.

### 18 Months Post Switch-On

#### Device Use and Benefit

As discussed earlier, a limitation of many studies of the effect of treatment of hearing loss with a hearing device is the lack of follow up and information regarding device use and the effectiveness of the devices, without which it is not possible to examine the relationships between the treatment and its effects. In the current study, average CI usage of 14 h per day was reported, along with significant mean improvements in speech perception in both quiet and noise at 18 months post switch-on. Only one participant did not improve in their speech perception ability post cochlear implantation. Study participants also reported a threefold decrease in listening disability in their everyday lives, and there was a statistically significant improvement in their ability to conduct conversations. These benefits are similar to those reported in the only other longitudinal study of cognition in older adults after cochlear implantation (Mosnier et al., 2015, 2018).

#### Cognition Outcomes

At 18 months post switch-on, there was no significant decline in group mean performance on any cognitive test, apart from a trivial (less than 1% of the baseline mean) negative change in the average visual attention (IDN) score. However, the mean executive function (GMLT) score increased by 18% of the baseline mean score, although this improvement was not statistically significant at the 5% level, (*p* = 0.097) in this small sample. Further exploration of this result using regressions showed that males who did not have higher education had significantly improved executive function (GMLT) by almost one third of the baseline mean score. The average executive function (GMLT) score for males with higher education and also for females (only one female had higher education, so this factor was not examined separately for females) did not change significantly, although there was a non-significant improvement in average score in both groups.

While cochlear implants have been shown to significantly improve speech perception (Dowell, 2012; Lazard et al., 2012; Blamey et al., 2013), mood, social isolation, function and quality of life in adults (Francis et al., 2002; Mo, 2005), until 2015 there were no studies in the English literature that prospectively examined the effect of CIs on cognitive function in older adults (Miller et al., 2015). Since then, three studies have evaluated cognitive function in CI recipients. The first two studies evaluated the same group of patients aged 65 and older at baseline at two postoperative intervals (*N* = 94 and 70, respectively). A battery of tests to evaluate episodic memory, visuospatial abilities, attention span, processing speed, mental flexibility and executive function was used pre-operatively, at 6 and 12 months post-operatively (Mosnier et al., 2015), and again 7 years after cochlear implantation (Mosnier et al., 2018). Cognitive scores were not treated as continuous data, but were instead categorized as normal or abnormal, with the number of normal/abnormal test outcomes documented for each participant. At 1 year post cochlear implantation, group mean scores had improved across all cognitive domains for 80% of the participants with the poorest pre-operative scores, while for patients with normal cognitive pre-operative performance, scores for memory, attention and executive function did not change significantly. It was not possible to evaluate change in verbal fluency scores, as the stimulus words were changed at each test session to avoid practice effects. The degree of improvement in cognitive performance for the poorest participants in the Mosnier et al. (2015) study at 1 year after implantation appears greater than that observed in the current study at 18 months post-switch-on. However, at baseline in the Mosnier study, many of the participants were functioning at a lower level cognitively than those in the current study. Half of the participants in the Mosnier et al. (2015) study had mild cognitive impairment at baseline, and 14% did not achieve a score in the normal range on the MMSE, whereas 100% of the participants in the current study did. It should be noted that executive function in the Mosnier study was measured using the Trail Making Tests (versions A and B), which have only poor (version A; 0.53) to moderate (version B; 0.67) test-re-test reliability, and are subject to significant practice effects over time periods of less than a year (participants in the first study were tested at 6-month intervals after cochlear implantation). These factors may have affected the cognitive results obtained in these studies. Further, the use of written test instructions for tests that were designed to be presented verbally in order to avoid over-diagnosis of cognitive impairment due to participants misunderstanding instructions may also have affected the results. For several of the cognitive tests used, changing the modality of test administration changes the cognitive task, and may confound the test results.

The third study (Jayakody et al., 2017b) evaluated 16 CI recipients 1 year after cochlear implantation using a non-verbal computer-based assessment battery (CANTAB; Cambridge Cognition Ltd., 2012). As in the current study, participants showed significantly improved speech perception in quiet (performance in noise was not measured) and performed significantly better on working memory, attention and processing speed, and some measures of executive function. Depression, anxiety and stress scores also significantly decreased. Other risk factors for cognitive decline were not measured or controlled for, the statistical analysis comprising *t*-tests and correlation analyses.

#### Quality of Life

Unsurprisingly, the benefits to audition and speech perception conferred by a CI were accompanied by significantly improved HUI-3 HRQOL outcomes for overall self-rated HRQOL and also for hearing disability for females at 18 months post switch-on. HRQOL did not improve significantly for males, but their baseline HRQOL scores were already higher than those for females. Given females are often more sociable than males, it is possible that the females in this sample were more affected by the social limitations of having a severe-profound hearing loss than the males, and this may have contributed to their lower self-reported QOL. Significant improvements in these domains have been reported previously in many studies of cochlear implant recipients, both male and female (e.g., Cohen et al., 2004; Damen et al., 2007). The significant association between better QOL and better executive function observed at baseline was sustained post-operatively, and higher QOL was also significantly correlated with better psychomotor function and also with a greater CogState composite Processing Speed score. It was also interesting to see that older participants reported larger reductions in their hearing disability after receiving a CI, although there is no obvious reason for this.

Mosnier et al. (2015) also reported significant improvements in QOL at 6 months post implantation on all six subdomains of the Nijmegen Cochlear Implant Questionnaire (Hinderink et al., 2000; NCIQ). In longer-term follow up, there was no significant change in scores on any sub-domains between 6 months and 7 years after cochlear implantation however (Mosnier et al., 2018). Although higher QOL was correlated with higher speech perception scores 1 year after implantation, the effect of QOL on cognitive outcomes was not investigated.

### Strengths and Limitations of This Study

Currently, the number of participants in this study is small, particularly in the group assessed 18 months after cochlear implantation, but this will grow over time as recruitment and further follow-up continues. The study has several significant advantages over previous studies of the effects of hearing devices on cognition in older adults, in particular the use of the Cogstate cognitive assessment battery, which is one of few suitable for people with severe-profound hearing loss, is comprehensive, not susceptible to practice effects, highly reliable and sensitive to change in function. Further strengths of the study are repeated audiometric assessment of hearing loss, documentation of device use (both objectively and subjectively), and assessment of many other risk factors for cognitive decline (e.g., biomarkers [the APOE 4 allele], social isolation, loneliness, education, mood, activity, quality of life, diet, and medical health), most of which were not included in the studies of Mosnier and colleagues, or in many other studies, and which will facilitate the analysis of the interactions of these factors and the device on cognitive outcomes. The inclusion of this comparison group will enable rates of cognitive decline to be compared between the cochlear implant recipients with older adults in the general community, and to determine whether long term cognitive function is related to the CI treatment of hearing loss or to a natural trajectory of decline in cognitive function in older adults.

## Conclusion

Despite the fact that these are initial results based on a small sample size, increased hearing loss and age were found to predict significantly poorer executive function and visual attention prior to cochlear implantation, while tertiary education predicted better executive function. Eighteen months after cochlear implantation, in addition to the expected benefits to speech perception, communication and QOL, significant improvements in executive function were observed for non-tertiary educated males, while cognitive function did not decline for the other participants. Long-term data collected at 18 month intervals with a larger sample of CI recipients and compared with that of the comparison group of healthy aging adults without severe-profound hearing loss will enable the effects of treatment of hearing loss with CIs on all outcomes to be evaluated, and whether this intervention can delay or even partially reverse the onset of cognitive decline.

## Ethics Statement

This study was carried out in accordance with the recommendations of the Australian National Health and Medical Research Council. All participants gave written informed consent in accordance with the Declaration of Helsinki. The protocol was approved by the Human Ethics Committee of the Royal Victorian Eye and Ear Hospital (Reference No. 15/1217H). There were no additional considerations.

## Author Contributions

JS, DH, PM, and PB contributed to the conception and design of the study. PB and JS designed the database. PB programmed the database and extracted the data. DH performed the statistical analysis and wrote the section “Results.” JS wrote the first draft of the manuscript and subsequent revisions. All authors contributed to the manuscript revision, and read and approved the submitted version. JS and DH revised the submitted version.

## Conflict of Interest Statement

The authors declare that the research was conducted in the absence of any commercial or financial relationships that could be construed as a potential conflict of interest.
